# Halophytes as new model plant species for salt tolerance strategies

**DOI:** 10.3389/fpls.2023.1137211

**Published:** 2023-05-11

**Authors:** Anita Mann, Charu Lata, Naresh Kumar, Ashwani Kumar, Arvind Kumar, Parvender Sheoran

**Affiliations:** ^1^ ICAR-Central Soil Salinity Research Institute, Karnl, Haryana, India; ^2^ ICAR-Indian Institute of Wheat and Barley Research, Shimla, Himachal Pardesh, India; ^3^ Department of Biochemistry, Eternal University, Baru Sahib, Himachal Pardesh, Ludhiana, India; ^4^ ICAR-Agriculture Technology Application Research Center, Ludhiana, India

**Keywords:** halophytes, transcriptomics, salinity, DEGs (differentially expressed genes), gene transformation, salt tolerance, osmoregulation

## Abstract

Soil salinity is becoming a growing issue nowadays, severely affecting the world’s most productive agricultural landscapes. With intersecting and competitive challenges of shrinking agricultural lands and increasing demand for food, there is an emerging need to build resilience for adaptation to anticipated climate change and land degradation. This necessitates the deep decoding of a gene pool of crop plant wild relatives which can be accomplished through salt-tolerant species, such as halophytes, in order to reveal the underlying regulatory mechanisms. Halophytes are generally defined as plants able to survive and complete their life cycle in highly saline environments of at least 200-500 mM of salt solution. The primary criterion for identifying salt-tolerant grasses (STGs) includes the presence of salt glands on the leaf surface and the Na^+^ exclusion mechanism since the interaction and replacement of Na^+^ and K^+^ greatly determines the survivability of STGs in saline environments. During the last decades or so, various salt-tolerant grasses/halophytes have been explored for the mining of salt-tolerant genes and testing their efficacy to improve the limit of salt tolerance in crop plants. Still, the utility of halophytes is limited due to the non-availability of any model halophytic plant system as well as the lack of complete genomic information. To date, although *Arabidopsis* (*Arabidopsis thaliana*) and salt cress (*Thellungiella halophila*) are being used as model plants in most salt tolerance studies, these plants are short-lived and can tolerate salinity for a shorter duration only. Thus, identifying the unique genes for salt tolerance pathways in halophytes and their introgression in a related cereal genome for better tolerance to salinity is the need of the hour. Modern technologies including RNA sequencing and genome-wide mapping along with advanced bioinformatics programs have advanced the decoding of the whole genetic information of plants and the development of probable algorithms to correlate stress tolerance limit and yield potential. Hence, this article has been compiled to explore the naturally occurring halophytes as potential model plant species for abiotic stress tolerance and to further breed crop plants to enhance salt tolerance through genomic and molecular tools.

## 1 Introduction

Agricultural soil salinity, one of the major abiotic stresses, affects crop productivity worldwide with a monetary loss of approximately 806.4 billion rupees per year (Qadir et al., 2014). Various soil, crop, and environmental factors are responsible for afflicting salinity, thereby limiting global food production for the ever-increasing human population. Land degradation including the changing climate, water scarcity and increased dependence on poor water quality, injudicious fertilizer use, and agricultural pollution are the major concerns toward salt enrichment of soil and water. More importantly, faulty management practices often contribute toward turning productive agricultural landscapes into wastelands due to higher accumulation of calcium, magnesium, and sodium along with anions such as sulfates and carbonates. All these factors limit water availability in the soil and its absorption by growing plants, thus creating a physiological drought. All over the world, nearly 20% of cultivated land is affected by soil salinization (Machado and Serralheiro, 2017), which is predicted to expand up to 50% by 2050 (Kumar and Sharma, 2020). In India, 6.73 million ha of lands are salt-affected constituting approximately 40% saline and 60% sodic in nature with a projected expansion of these lands up to 11 and 16.2 Mha by 2030 and 2050, respectively (CSSRI Vision 2050, https://www.cssri.org). The soils containing more sodium salts with electrical conductivity of the saturated paste extract (ECe) more than 4 dS m^−1^ are characterized as saline soils. On the other hand, sodic soils have more carbonates and bicarbonates, thereby increasing the alkalinity in the soil (soil pH > 8.5). Crop irrigation with high RSC water also increases soil pH over a period of time, thereby reducing rice and wheat productivity by 16% and 14%, respectively (Sheoran et al., 2021a). In such conditions, soil ameliorants are used for improving physiological traits, thus helping plants to survive under abiotic stress. The effect of salinity stress on plant growth and development is considerable, affecting seed germination, plant anatomy and physiology, root architecture, protein synthesis, gene expression, and fructification. Plants grown in saline environments first experience osmotic stress and later imbalanced ion homeostasis, and these two stresses cumulatively cause oxidative stress and a sequence of other secondary stresses affecting plant metabolism (Kumar et al., 2019; Kesh et al., 2022).

For most plants, specifically the glycophytes, salinity is a major constraint limiting plant growth and, consequently, current and future agricultural production. To adapt to such situations, the easiest solution may be the selection of plants that can naturally grow and survive in extreme saline habitats, i.e., halophytes which have been explored scientifically since the early 20th century (Stocker, 1928). Many studies provide insights into the physiological mechanisms of tolerance of halophytes (Colmer and Flowers, 2008; Flowers and Colmer, 2008) as well as their potentiality for agriculture and ecological services (Rozema et al., 2013). Given the widespread changes and increased degradation in salinity-affected drylands and coastal areas, the need for enriching knowledge and technical know-how concerning halophytes and the related database will undoubtedly gain momentum in the coming years. The fact that some plants can tolerate and survive higher salt concentrations which otherwise kill most other species is an interesting phenomenon to begin with. However, salt tolerance in the plant kingdom is much more than just a scientific curiosity since most of our crop species fall into the “sensitive” category, and the areas suitable for their cultivation are shrinking day by day as a result of increased desertification and salinization. Although many salt-tolerant varieties are being cultivated over different regions and various climate-resilient management strategies have been proposed for different ecosystems (Sheoran et al., 2021b; Sheoran et al., 2022), consequently, understanding the eco-physiological mechanisms and the biogeography and ecology of salt tolerance in higher plants and their associated microorganisms is a vital step toward generating salt-tolerant plant types. The use of potential halophytes in phytoremediation of salt-affected areas and in the ecological restoration and rehabilitation of degraded ecosystems may emerge as being, or more, important than biosaline agriculture itself, and halophytes are being explored for desalination of saline lands. A new concept of “circular halophytes mixed farming (CHMF)” is also emerging using halophytes along with crop plants to develop a soil–water–plant salinity dynamics model for stimulation of the cultivation and management strategies through long growing seasons. This model is based on the concept of ion sequestration from the root zone of crop plants through halophytes for sustainable crop productivity (Toderich et al., 2022).

The polygenic nature of salt tolerance makes it complex and difficult to improve crop salt tolerance through conventional breeding for selection. Although quantitative trait loci (QTL) analysis of multiple intervarietal crosses and their pyramiding has been identified, this cannot be expected to develop plant types with the desired salt tolerance levels comparable to halophytes. There always remains a possibility for a low success rate presumably due to differences in the genetic makeup of the donor and recipient species and their interaction with environmental factors. As an alternative, genetic engineering is considered to be a promising strategy for improved salt tolerance. However, a proper selection of candidate transgenes requires a detailed knowledge of the molecular mechanisms of inherent salt tolerance in halophytes. Gene transfer from a “related and co-existed species” to crop plants can be a possible alternative for the genetic engineering of crop plants having improved salt tolerance with a high success rate. Salt-tolerant grasses (STGs) and weeds could be potential sources for different candidate genes for engineered plant types for various abiotic stressors on account of their hardiness, relatedness, and co-existence with crops. It is likely that the genes that are responsible for the superior salt tolerance in halophytic grasses may serve as a subset of the genes for improvement in crops. Therefore, further exploration is needed to test the contribution of salt stress-related genes or regulatory factors from highly salt-tolerant halophytes for their possible utilization in crop improvement. Although many halophytes such as *Sueda* (Guo et al., 2019), *Spartina* species (Baisakh et al., 2008; De Carvalho et al., 2013), *Salicornia brachiata* (Jha et al., 2009), *Atriplex*, *Dichanthium* (Mann et al., 2019a), *Sporobolus* (Mann et al., 2019b), *Urochondra* (Mann et al., 2021b), *Salvodora* (Kumari and Parida, 2018; Kumar et al., 2022), *Aegilops tauschii* (Mansouri et al., 2019), *Amaranthus*, and *Chenopodium* species are being explored for their salt tolerance mechanisms and utilization as gene donors for enhanced salt tolerance in crop plants, still, *Arabidopsis thaliana* and *Thellungiella halophila* (salt cress) have been used as model halophytic plant species for functional validation in most salt tolerance studies. The major limitation is the non-availability of complete genomic information on these halophytes; hence, this puts constraints on using these halophytes as donors for abiotic stress studies. Hence, in this article, an attempt has been made to briefly compile the proposed mechanisms in salt-tolerant halophytes for genetic improvement of salt tolerance in agricultural crop plants including the limiting factors for their potential use.

## 2 Behavior of halophytes in saline conditions

Plants naturally growing in saline environments called “halophytes” can be defined as a group of plants surviving and completing their life cycle in environments with a salt concentration of 200 mM or more (Flowers and Yeo, 1986). These halophytic plants occupy ∼1% of the entire flora present on the earth’s surface (Kumari et al., 2015). The halophyte database eHALOPH (http://www.sussex.ac.uk/affiliates/halophytes/) identifies more than 1,500 species as salt-tolerant plants (Santos et al., 2016) based on their adaptive anatomical, physiological, or morphological features in highly saline soils. Halophytes have also been categorized based on their salt preferences like obligate or facultative halophytes and further subcategorized as xerophytes, mesophytes, succulents, etc. The initial database of halophytes, HALOPH, was assembled by Aronson (1989) and contained 1,560 plant species in 550 genera and 117 families. Le Houerou (1993) further enriched Aronson’s list including 5,000–6,000 species or 20%–30% of the terrestrial halophytic flora, contributing to 2% of the world’s angiosperm species. The potentiality of halophytes to outlive under extremely harsh conditions is due to their built-in mechanisms including ion compartmentalization, osmotic adjustments, succulence, antioxidative defense systems, redox homeostasis and accumulation/removal of salts, and the expression of genes involved in these pathways (Flowers and Lauchli, 1983). In general, 500 mM of Na^+^ and Cl^−^ contents remain in the cell flow of most of the halophytic species, although an extreme intracellular Na^+^ of 2000 mM concentration has also been reported in *Tecticornia* to maintain a positive turgor pressure for osmotic adjustments (Flowers et al., 2015) as against a cellular concentration of 1–10 mM of Na^+^ and 100–200 mM of K^+^ in higher plants growing under normal (non-saline) conditions. It is interesting to note that the adaptive compatibility of these halophytes is not only because they are salt-loving but also mainly because they have better ionic balance and compartmentalization than other plants growing in non-saline habitats. Additionally, better physiological capacity under higher saline environments *via* higher transpiration, shallow root architecture, and a tendency to exudate the dissolved salts has also been reported by Kumar et al. (2019).

**Figure 1:**
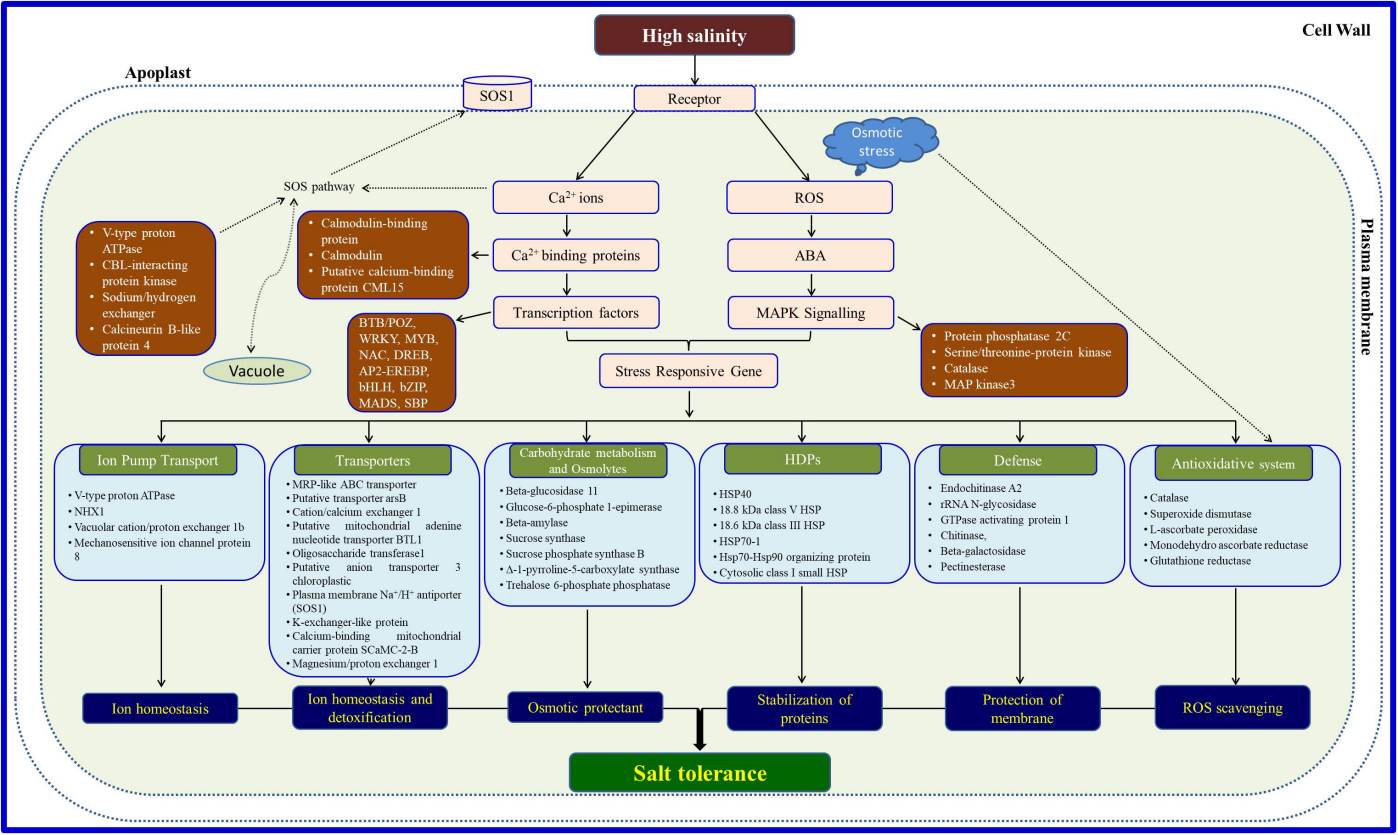
Possible tolerance and survival strategies operative in halophytes under salinity.

Salt tolerance, being a multigeneic trait, is mostly governed by more than one strategy (**Figure 1**) through structural changes including adaptive physiological and/or biochemical pathways such as discriminative uptake/accumulation or exclusion of specific ions, selective uptake of ions through transporters, tissue-specific compartmentalization of ions, accumulation of osmolytes, efficient gas exchange and reactive oxygen species (ROS) system, and upregulation of specific salt-responsive proteins or genes (Parida and Das, 2005; Mann et al., 2019a; Sankari et al., 2019). Salt stress initiation is accompanied first by the lowering of osmotic potential due to higher salt concentrations, releasing water out of the plant roots. As a result, glycophytes cannot withstand and die over a period of time, while halophytes are able to modify their certain mechanisms to adapt to such conditions enabling them to survive and flourish. Generally, most glycophytes can tolerate salts in water up to 125 ppm for survival, while halophytes have the capability to tolerate saline water in the range of 125–5,000 ppm, almost equal to seawater salinity. The second phase of salt stress commences with ionic stress, created mainly due to higher levels of Na^+^ and Cl^−^ ions disrupting plant metabolic function and, hence, plant survival. Broadly, halophytes follow two different strategies at high salt concentrations: salt tolerance, through the accumulation of salts in the cell, and salt avoidance, through physico-biochemical or morphological alterations for diluting salt accumulation in the plant cells or structural modifications to inhibit the entry of salts in the plant’s root system. Salt-accumulating halophytes maintain their water potential, being more negative than the external soil media since these plants keep on accumulating salts from the external saline environment which may lead to increased leaf thickness. The storage of these excessive salts is through some specialized structures such as salt glands on the leaf surface. Some salt accumulators avoid salt stress by minimizing salt concentration in the cytosol of their leaf cells. Leaf cells regulate cytosolic salt levels by transporting sodium and chloride ions into the central vacuole (Kumar et al., 2019). Mozafar et al. (1970) have reported better plant growth of *Atriplex halimus*, an extremely salt-tolerant Mediterranean species at different saline levels by tolerating much lower external water potential. Surprisingly, the salt concentration and water content in the leaves of *A. halimus* did not increase significantly under salinity excluding the role of osmotic adjustment (at least due to electrolytes) under salt stress. This shows that as the water content of the leaf tissue decreased, the cell sap did not get saturated with salts which apparently is transported to the vesiculated hairs on the leaf surface, while some other excluder plants shed their old leaves loaded with extra salt ions. These toxic ions move through the petioles and into the stem, and these petioles detach themselves from the stem due to heavy salt load; hence, extra toxic ions are removed from the plant stream. Some other plants try to avoid the entry of ions into the roots due to the ion-selective property of the cell membrane, but this mechanism is not far operative as the other ionic transporters are also located on the cell membrane. A few plant root cells pump out excessive sodium and chloride ions from the soil system. Anatomical modifications in the leaves also participate in salinity tolerance by increasing the thickness of the palisade parenchyma and decreasing the spongy parenchyma with the enlargement of intercellular spaces (Shiyab et al., 2013) helping in the gas exchange with reduced stomatal aperture.

## 3 Adaptive strategies of halophytes

ne ecosystems, where the majority of existing traditional crops do not survive. Outstandingly, there is no novel or unique trait in halophytes that is not possessed by traditional crop plants. As an alternative, halophytes adapted themselves by following “a bit better” strategy through a well-coordinated system to tackle salty ions; hence, salinity is not a stress factor for halophytes. The origin of halophytic plants shows different phylogenetic clades which indicate the polyphyletic evolution and widespread occurrence of halophytes, and hence, a single pathway cannot be traced or correlated with their survival strategy. Many reports show a homology of halophytic genes with that of glycophytes but with differential qualitative and quantitative expression levels (Bose et al., 2015). Most of the studies conducted on the salt tolerance mechanism of halophytes proposed that these organisms might adapt a number of adaptation strategies, for example, ion exclusion or inclusion, osmotic tolerance, and tissue tolerance which evidenced their supremacy over glycophytes in exhibiting salt tolerance. Mishra and Tanna (2017) validated salt stress-induced sensors leading to the initiation of stress tolerance mechanisms, involving various signaling proteins and up- or downregulation of numerous genes, and ending up with the individual or collective networks of stress-responsive genes. Starting from the onset of salinity, sensing, and signal transduction, downstream networking is associated with ABA-responsive stomatal movements, photosynthetic efficiency, water conservation, and many other alternative processes to cope with increasing ionic concentrations in plant cells. Here, we have compiled the major strategies adopted by halophytes for salinity tolerance. The major halophytes (salt tolerance of more than 300 mM of NaCl) with their adaptive features and strategies are listed in **Table 1**.

**Table 1:** Halophytic plants with their salt tolerance limit and adaptive strategies for survival.

Sr. no.	Name of the halophyte	Salt tolerance limit (mM NaCl)	Adaptive strategy	References
1.	*Aeluropus lagopoides*	350	Osmoprotectants, ROS scavenging, and ionic balance through K^+^ deposition	Kumar et al. (2016)
2.	*Atriplex lentiformis*	300	Low Na^+^/K^+^ in the roots	Kumar et al. (2021)
3.	*Atriplex nummularia*	500	Tissue-specific compartmentalization of ions, osmotic adjustment	Ramos et al. (2004)
4.	*Cakile maritima*	400	Enhanced activity of SOS system	Arbelet-Bonnin et al. (2018)
5.	*Dichanthium annulatum*	300-500	Restricted Na^+^ accumulation in the root zone	Kumar et al. (2018), Mann et al., (2019a)
6.	*Hordeum brevisubulatum*	700	Enhanced uptake of K^+^	Zhang et al. (2020)
7.	*Haloxylon salicornicum*	400	ROS scavenging and better osmotic adjustment	Panda et al. (2019)
8.	*Kosteletzkya virginica*	390	Upregulation of proline metabolism	Wang et al. (2015)
9.	*Leptachloa fusca*	500	Ion homeostasis and secondary metabolite accumulation	Lata et al. (2019)
10.	*Lycium humile*	750	ABA-mediated stomatal closure, water conservation, ion transport, and osmoregulation	Palchetti et al. (2021)
11.	*Pongamia pinnata*	500	Na^+^ sequestration and ion homeostasis	Marriboina et al. (2017)
12.	*Salsola drummondii*	500	Dilution of toxic salt ions through succulence mechanism	Elnaggar et al. (2020)
13.	*Salvadora oleoides*	350	Low Na^+^/K^+^ in the leaves	Kumar et al. (2022)
14.	*Salvadora persica*	750	ABA-mediated stomatal closure, water conservation, ion transport, and osmoregulation	Kumari and Parida (2018)
15.	*Scorzonera hieraciifolia*	600	Osmoregulation and ROS activity	Altuntas and Terzi (2021)
16.	*Spartina alterniflora*	600	Osmotic adjustment	Vasquez et al. (2006)
17.	*Sporobolus marginatus*	500	Better photosynthetic rate, maintaining root Na^+^ and K^+^ content, increased osmolyte accumulation	Kumar et al. (2018); Lata et al. (2019)
18.	*Suaeda nudiflora*	350	Salt hyperaccumulator, osmolytes, ionic balance through K^+^ deposition	Kumar et al. (2016)
19.	*Urochondra setulosa*	500-1,000	Salt excluder and ion sequestration, efficient photosynthesis, low membrane injury	Kumar et al. (2018); Mann et al. (2021b); Lata et al. (2022)
20.	*Zoysia macrostachya*	300	Osmoregulation and ROS activity	Wang et al. (2020)

### 3.1 Structural aberrations

Early plants originated in oceanic (high salinity) environments (Dansereau, 1957), but with time and evolution, plants have developed numerous adaptive mechanisms to cope with and sustain under saline conditions. Likewise, halophytes tend to have stilt or prop roots descending from the aerial parts to get hold of loose or muddy soil. Hydro-halophytes also modify their roots to negatively geotropic roots or breathing roots for aeration in oxygen-deficient soils. Such roots have the tendency to develop shoot-like characters with prominent aerenchyma enclosing large air cavities making a continuous connection with the cortex of the stems and the stomata of the leaves (Kumar et al., 2019). The inner layer of the endodermis in root cells develops waxy strips to prevent the transport of Na^+^ and Cl^−^ ions. The development of succulent stems with more cell elongation than cell division (Repp, 1961) by increasing pith and its cell area in the stem is also an adaptive strategy in halophytes to survive in response to surrounding ionic concentration under salt stress and to accumulate more water in hot environments. Similarly, the leaves of halophytes tend to reduce their surface area and become thicker and more succulent. Chang et al. (2022) observed decreased leaf area and increased leaf thickness in *Alhagi sparsifolia* with an increasing salt gradient. Recently, Iqbal et al. (2023) have observed structural and functional modulations in the facultative halophyte *Salvadora oleoides* Decne for adaptability under hyperarid and saline environments. The plants of the hypersaline desert flat ecotype had larger leaves with a thicker epidermis, while those from salt-affected and sandstone mountains had smaller leaves with a thin epidermis. Similarly, intensive sclerification with enhanced stomatal index was the main trait for adaptability in desert ecotypes. The ratio of palisade and spongy tissue in the leaves imparted plant adaptation toward increased salt content in *A. sparsifolia.* The tightness or looseness of palisade tissue in the leaves reflects the anatomical adaptation of spongy tissue in saline environments (Shiyab et al., 2013). The leaves become thin with only a green epidermis in submerged halophytes with the development of specialized trichomes under extremely arid conditions. Salt glands/bladders or epidermal bladder cells (EBCs) on young leaves also exist in most of the halophytes (Shabala et al., 2014; Kiani-Pouya et al., 2017) located for ion sequestration since these leaves have small vacuoles. In several halophytes, like *Avicennia marina*, *Avicennia officinalis*, *Suaeda salsa*, and *Suaeda maritima*, apoplastic channels in the roots act as barriers to Na^+^ ions providing salt adaptation (Rahman et al., 2021). A high vascular bundle/xylem ratio and vascular bundle/phloem ratio in the roots and leaves is also being developed in the ecotypes of warm and dry Cholistan Desert with small dunes for better translocation of sap toward the roots (Lechthaler et al., 2019). Tissue lignification is a critical response of plants to desiccation, as it protects the soft tissue from collapse and restricts water movement outside of the surface. In the aquatic halophyte *Fimbristylis complanata*, the formation of a thick sclerenchyma and collenchyma layer in parenchyma tissue was observed in salt-affected and sandstone mountain regions (Kaleem et al., 2022).

### 3.2 Physiological mechanisms

The physiological processes for salt tolerance in halophytes have been explored in the past. The very first physiological pathway being exploited for tolerance against salinity is osmotic adjustment through the accumulation of osmolytes and moving the inorganic ions including Na^+^ and Cl^−^ inside the vacuole. However, the toxic salts do not inhibit the growth of halophytic plants but affect the plants’ physiological processes indirectly. Ion compartmentalization leads to the accumulation of extra salt load in older leaves enhancing their shedding, thereby restricting further transport of photo-assimilates to the growing tissues (Munns et al., 1995). The imperative role of Na^+^/H^+^ antiporters in halophytes also discriminates them from salt-sensitive glycophytes although these channels are being expressed in salt-tolerant plants. Halophytes enter into the xeromorphic stage by absorbing and conserving available water, hence maintaining the balance between transpiration and water flow within the plant cells. The major physiological pathways adopted by halophytes are being discussed further.

#### 3.2.1 Selective uptake and transport of ions

**Figure 2:**
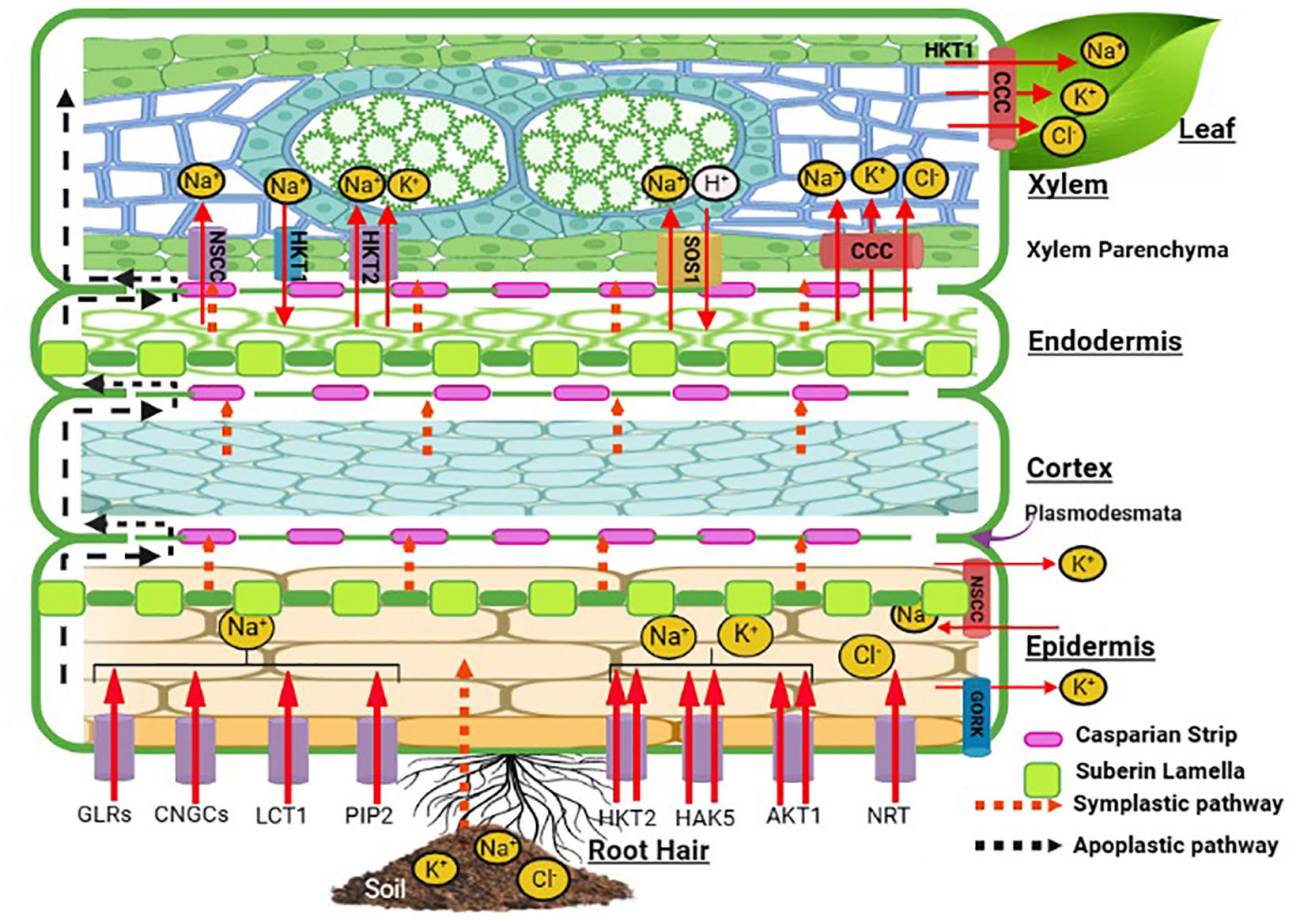
Schematic representation of ion uptake mechanism in halophytes. This figure is created with Biorender.com. AKT1, *Arabidopsis* K^+^ transporter 1; CCC, cation chloride cotransporter; CNGC, cyclic nucleotide-gated channel; GORK, gated outwardly rectifying K^+^ channel; GLR, glutamate receptor-like channel; HKT, high-affinity potassium transporter; HAK, high-affinity potassium uptake transporter; LCT, low-affinity cation transporter; NRT, nitrate transporter; NSCC, non-selective cation channel; PIP2, plasma membrane intrinsic protein; SOS1, salt overly sensitive-1.

All halophytic plants have a common regulatory control for Na^+^, Cl^−^, and K^+^ uptake and transport against external water potential. Halophytic plants acclimatize themselves in saline habitats through acquired mechanisms that permit selective uptake of K^+^ in the face of significant competition from a blend dominated by Na^+^ ions. However, the situation is not simply the affinity for highly selective K^+^ uptake, but halophytes also tend to exploit Na^+^ for osmotic adjustments, although to different degrees in contrasting situations. As a coping mechanism, halophytes have developed specific anatomical and morphological features which regulate the entry and localization of these preferential and toxic ions through tissue- and organ-specific ion compartmentalization (Flowers and Colmer, 2008). Following this, the multifaceted mechanism of osmotic adjustment gets started. A radial transport and loading of Na^+^ and K^+^ into the root xylem is followed by a compound cascade of reaction for Na^+^ sequestration in vacuoles in the roots (**Figure 2**) and leaf cells, ion transport in guard cells, control of ion fluxes into salt glands and bladders, and oxidative signaling and damage repair inside the plant cells. Ionic movement in the roots takes place *via* symplastic or apoplastic pathways. The root epidermal layer has membrane transporters for Na^+^, K^+^, and Cl^−^ such as HKT2, AKT, NRT, PIP2, LCT1, and CNGCs. HKT, HAK, and AKT transporters show specificity for K^+^ excluding Cl^−^ ions out of anionic uptake competition. Suberin lamellae in the epidermis may act as a barrier for Na^+^ influx into the roots with further ion selectivity at the Casparian strips. The movement of ions through the cortex and endodermis continues to the xylem with passive movement through the NSCC, and Na^+^ ions are loaded *via* SOS1 and HKT2. HKT1 translocates Na ions into the xylem parenchyma. These ions are further translocated to upward plant tissues *via* a shoot transpirational stream for compartmentalization or ion homeostasis. Relatively high rates of net Na^+^ uptake are sustained by halophytes ranging between 1 and 10 nmol Na^+^ g^−1^ fresh root mass without injury to the plant. Na^+^ influx in the halophyte *Spergularia marina* showed that uptake of Na^+^ was linear over 2 h in a “steady-state” salinity. At 25-500 mm of NaCl, the Na^+^ influx rate was 2–13 nmol/g root fresh mass over 24 h in halophytes (Yeo, 1974), while it was 30 nmol g^−1^ root fresh mass in just 50 mm of external NaCl in *Arabidopsis* (Flowers and Colmer, 2008). The comparative ionic distribution in *Suaeda nudiflora*, *Suaeda fruticosa*, *Portulaca oleracea*, *Atriplex lentiformis*, *Parkinsonia aculeate*, and *Xanthium strumarium* under different salinity levels also showed the selectivity of ionic transporters for K over Na along with Ca and Mg ions (Devi et al., 2016). In the halophyte grasses *Aeluropus* and *Sporobolus* growing at the salinity level of 83.06 dS m^−1^, the inhibitory effect of high soluble salt content in soil was observed through reduced uptake of Na^+^ and Cl^−^ with more uptake of K^+^, Ca^2+^, Mg^2+^, and SO4^2−^ (Mangalassery et al., 2017). The tissue preference of Na^+^ and K^+^ accumulation was favored more in the roots than in the leaves of *Glycyrrhiza inflata* at 50 and 200 mM of NaCl (Cai et al., 2023) and of *Sesuvium portulacastrum* at 150-500 mM of NaCl showing ion selectivity of the membrane transporters HKT1 and 2, NHX1, NHX3, vATPase, and SOS1 (Nikalje et al., 2018).

#### 3.2.2 Ion regulation and compartmentalization

Generally, ionic homeostasis is disturbed in the plant cells as result of abiotic stress. Therefore, the uptake and compartmentalization of ions is quite critical for plant growth and development not only in favorable environments but also under saline conditions. For all plants, either glycophytes or halophytes, large quantities of salt accumulation in the cytoplasm are detrimental. Consequently, halophytes either restrict the entry of excessive salts into the vacuoles or compartmentalize the ions in different plant parts by maintaining ionic equilibrium. The vacuolar membrane sodium potassium antiporter proteins on the tonoplast are the key players in the management of Na^+^ and Cl^−^ ions involving primarily two energy-dependent hydrogen pumps, H^+^-ATPase (V-ATPase) and pyrophosphatase (VPPase). Since approximately 80%–95% osmotic pressure of cell sap is due to the presence of inorganic ions, Na^+^, Cl^−^, and K^+^, in the cytosol, hence, these ions play a pivotal role in maintaining the shoot osmotic and turgor pressure in halophytes under saline conditions (Storey, 1995; Shabala and Mackay, 2011) which is generally achieved through *de novo* synthesis of compatible solutes in glycophytes. In *Urochondra setulosa*, a highly salt-tolerant halophytic plant, higher Na^+^ accumulation in the roots was observed at the salinity level of ECe ~40 dS m^−1^ (Lata et al., 2019) without any reduction in K^+^ content. In *Aeluropus lagopoides* and *S. nudiflora*, the accumulation of Na^+^ and Cl^−^ ions increased in the roots as well as in the shoots under stress conditions (Kumar et al., 2016). Similarly, in *S. salsa*, V-ATPase activity was upregulated than V-PPase under salt stress (Wang et al., 2001). The vacuolar sequestration through H^+^-ATPases and H^+^-pyrophosphatases energizes the tonoplastic membrane, and the Na^+^/H^+^ exchangers (NHX) create the Na^+^ influx into the vacuole against H^+^. At extreme salinity, it becomes imperative to exclude the high content of Na^+^ ions since these ions accumulate in the plant cells. This leads to the activation of Ca^2+^ signaling and Na^+^ ion sequestration through the SOS detoxification pathway (Gupta and Huang, 2014). This *SOS1* protein also helps in Na^+^ loading into xylem tissues and, hence, regulates the ion homeostasis in the root–shoot transpirational stream. Higher accumulation of Na^+^ in the roots followed by the stems in both *Aeluropus* and *Sporobolus* showed that the translocation of Na^+^ ions to the leaves is prohibited to reduce the adverse impact of these toxic ions along with the increased accumulation of K^+^, Ca^2+^, and SO_4_^2^ ions (Mangalassery et al., 2017). In *Lycium ruthenicum*, the distribution ratio of Na^+^ and K^+^ in various tissues also changed with the addition of an appropriate amount of salt (100-450 mM of NaCl) where the Na^+^/K^+^ ratio was lower in the roots but higher in the shoots (Hu et al., 2022).

#### 3.2.3 Compatible solutes and osmoregulation

One of the important criteria for a tolerant cell model toward salt tolerance is the higher levels of compatible solutes in the cytoplasm to counterbalance the more negative osmotic potential across tonoplast due to Na^+^ and Cl^−^ ions in the vacuole (Wyn and Gorham, 2002). Due to the influx of high concentrations of salt ions, osmotic pressure is severely compromised under salt stress. Under abiotic stress, cation uptake is generally more than the anion which creates the potential difference across the membrane. Excessive salt ions need to be transported to the cell for osmoregulation; hence, the energy trade-off between salt tolerance and growth is set up preferring tolerance. In such situations, plants tend to synthesize or overexpress organic compounds like soluble sugars, amino acids, alcohols, and polyols under stress conditions. These compounds help in maintaining the turgor pressure, hence the cell volume and fluid balance in the cell for its normal functioning (Greenway and Munns, 1980). Some halophytes (*Zygophyllum xanthoxylum* and *S. portulacastrum*) absorb more Na^+^ and use it for osmotic adjustment in arid environments (Slama et al., 2007; Ma et al., 2012). For example, the enhanced uptake of K^+^ plays a key role in osmoregulation under stress conditions in a few halophytes. Similarly, β-alanine betaine accumulation is limited in a few plant species, while the amino acid proline accumulates predominantly in taxonomically different sets of plants including halophytic plant species. The xero-halophyte species *A. halimus* L., collected from two locations, i.e., from a salt-affected coastal site (Monastir) or from a non-saline semiarid area (Sbikha) in Tunisia, differed in their selectivity for the accumulation of proline or glycine betaine in response to salinity and water stress. Interestingly, the major osmoprotectant under saline conditions (160 mM NaCl) was glycine betaine, while under water stress (15% PEG), proline accumulated more (Hassine et al., 2008). The accumulation of the proline analog 4-hydroxy-N-methyl proline (MHP) and dimethyl-sulfonioproprionate (DMS) was reported in *Melaleuca bracteata* and *Spartina* spp., respectively (Naidu et al., 2000). The accumulation of osmoprotectants within the cell is sustained either by an irreversible synthesis of the compounds or by a combination of synthesis and degradation. With salt stress, the expression levels of betaine aldehyde dehydrogenase (BADH) and choline monooxygenase (CMO) increased in *S. maritima*, *Salicornia europaea*, *Suaeda aegyptiaca*, and *Atriplex nummularia*, thereby promoting glycine betaine synthesis (Askari et al., 2006). Higher levels of glycine betaine and raffinose were reported to enhance salt tolerance (Wu et al., 2010). The accumulation of organic acids, sugars, sugar alcohols, proline, and betaine was reported in *Haloxylon salicornicum*, *Puccinellia tenuiflora*, *Salvadora persica*, and *Glycyrrhiza inflata* for osmotic adjustment (Guo et al., 2010; Kumari and Parida, 2018; Panda et al., 2020; Cai et al., 2023). In a halophytic perennial grass (*Sporobolus marginatus*), osmotic adjustment through the accumulation of proline, total soluble sugars, glycine betaine, and total proteins helped in proper plant growth and functioning up to salinity stress of EC 35 dS m^−1^ and alkalinity of pH 10.0 (Mann et al., 2019b). Similarly, proline accumulation was approximately 5-10 times higher in mixed saline–sodic conditions of pH 10.0, ECe 35 dS m^−1^ and of pH 9.0 + ECe 20 dS m^−1^, respectively, in the halophytes *S. marginatus* and *U. setulosa* (Kumar et al., 2019).

#### 3.2.4 Scavenging of reactive oxygen species

Generally, most of the reactive oxygen species (^1^O_2_, O^2−^, OH^−^, and H_2_O_2_) generated in every plant cell from various metabolic processes act as signaling molecules in various physiological mechanisms (Hassan et al., 2017). Excessive levels of ROS in the cells degrade DNA, proteins, and lipids and consequently disturb the activities of the photosynthetic apparatus, cellular metabolic enzymes, and cell membrane integrity (Yu et al., 2011). In the plant cell, a steady-state balance between the antioxidant scavenging system and the accumulation of ROS enables adequate redox biology and normal plant growth regulation processes. For scavenging the excess ROS/toxic radicals, plants produce various types of antioxidant enzymes such as superoxide dismutase (SOD), catalase (CAT), dehydroascorbate reductase (DHAR), ascorbate peroxidase (APX), thioredoxin reductase (TRX), Peroxiredoxin (PRX), glutathione peroxidase (GPX), and glutathione S-transferase (GST). Based on their activity, these enzymes may be categorized as enzymatic and non-enzymatic. Enzymatic antioxidants break down the free radicals (dangerous oxidative products) into hydrogen peroxide and then into water through multiple chain reactions in the presence of cofactors such as boron (B), zinc (Zn), chloride (Cl), iron (Fe), manganese (Mn), nickel (Ni), copper (Cu), and molybdenum (Mo) and remove the free radicals from the cells. Conversely, the non-enzymatic antioxidants such as glutathione, vitamin C, plant polyphenol, vitamin E, and carotenoids interrupt the multiple chain reactions of free radicals and help the plant to counteract the negative effects of high salt stress in performing regular processes (Yu et al., 2011; Das and Strasser, 2013; Wang et al., 2013; Bose et al., 2014). Importantly, the enzymes SOD, CAT, and GPX having a large molecular structure absorb the free radicals (ROS) and prevent ROS from attacking the other important proteins in the cell. Notably, SOD is also known as a first line of defense against biotic or abiotic stresses. Usually, the generation of ROS species and the toxicity mechanisms are similar in glycophytes and halophytes; however, the strategies for detoxification and prevention differ in terms of total antioxidant activity and isoenzymatic form expressed in response to salinity. With the onset of abiotic stress, these ROS can stimulate various Na^+^ and K^+^ membrane ion channels required for maintaining optimum cytosolic K^+^/Na^+^ ratios imparting salt tolerance (Anschütz et al., 2014). During ROS scavenging, the enzyme activities of DHAR, MDHAR, APX, and GR determine the AsA–GSH pathway along with the reduced form of AsA and GSH antioxidants, enhancing the salt stress tolerance in plants (Kohli et al., 2019). SOD plays an important role to define the specific H_2_O_2_ “signature” in halophytes, and hence, its higher intrinsic activity triggers a cascade of physiological and molecular responses for stress signaling (Bose et al., 2014). Proteome-level changes in *Halogeton glomeratus* revealed the abundance of antioxidative enzymes, viz., Fe-superoxide dismutase (FeSOD), cytosolic APX, and phospholipid hydroperoxide glutathione peroxidase (PHGPX), under salt conditions regulating the balance of ROS formation and removal to defend against oxidative stress and cell damage (Wang et al., 2014). Similar reports on other halophytes such as *S. fruticosa* (Bankaji et al., 2016), *Limonium delicatulum* (Souid et al., 2018), *S. marginatus* (Mann et al., 2019b), *H. salicornicum* (Panda et al., 2019), and *Atriplex canescens* (Feng et al., 2023) also revealed enhanced antioxidant enzyme activities under salinity stress highlighting the stronger capability of these halophytes in removing ROS and protecting themselves from oxidative damage and maintaining redox homeostasis.

## 4 Genetic regulation of salt tolerance in halophytes

The growing habitats of halophytes vary from saline wastelands to coastal ecosystems, various salt-affected ecosystems (coastal and inland), flat salt lands, and steppes. During evolution, adaptive strategies have been intrinsically developed in halophytes with time to tolerate salinity stress up to EC 50 dS m^−1^ (~500 mM of NaCl) (Kumar et al., 2019) or almost equal to seawater (Shabala, 2013). Briefly, based on the available literature and our previous studies, we have compiled the tentative salt tolerance mechanisms in halophytes. The advancements in bioinformatics and molecular biology techniques have successfully decoded the physiological, biochemical, genetic, proteomic, and transcriptomic coordination and regulation of the tolerance mechanisms in many halophytic plants, and the draft genomes of a few halophytes, e.g., *Thellungiella parvula* and *Thellungiella salsuginea*, are also available in the database (Dassanayake et al., 2011; Wu et al., 2012).

The major halophytic genes responsible for salt tolerance are cation/proton antiporters on the vacuolar membrane (NHX) including H^+^-ATPases and vacuolar H^+^-pyrophosphatase, as well as transporters on the plasma membrane (SOS gene network)—potassium transporters. Apart from these, genes for several other biomolecules such as osmolytes and antioxidant enzymes and for specific modified plant structures in halophytes such as bladders and salt glands are also expressed, helping the plants to survive under salt stress conditions. Salt sensors get triggered at the onset of salt stress, activating the SOS pathway-related genes and linking multiple signaling associates. Thereafter, an individual or interactive consequence of the identified genes might promptly activate the defense mechanism through ionic homeostasis and ROS detoxification. Although halophytes possess a unique salt tolerance mechanism (**Figure 1**) over glycophytes, primarily, common genes are involved in metabolic pathways for imparting tolerance in both halophytes and glycophytes.

### 4.1 Genes encoding ion compartmentalization and homeostasis

A number of genes are associated with the regulation of ionic uptake and transport in halophytes governing the salt exclusion and compartmentalization (SOS1, SOS2, SOS3), osmoregulation (NHX, V-type and P-type H^+^-ATPases, H^+^-pyrophosphatase), photosynthesis, ion transporters, ROS system, etc. (Mann et al., 2021a). These genes help in the regulation of ionic influx and efflux, and their expression generally remains higher in halophytes in comparison to glycophytes. The *SOS1* gene encodes the plasma membrane Na^+^/H^+^ antiporters, which are responsible for sodium exclusion from the apoplast. Alternatively, the *SOS2* gene encodes the serine/threonine type protein kinase, and the *SOS3* genes encode an EF-hand-type calcium-binding protein, which regulates Na^+^ and K^+^ transport. Recently, two more components, SOS4 (pyridoxal kinase) and SOS5, have also been reported, where SOS4 is involved in pyridoxal-5-phosphate (vitamin B_6_) biosynthetic pathway and Na^+^/K^+^ homeostasis, while SOS5 is a putative cell surface adhesion protein, required for normal cell expansion (Saddhe et al., 2020). H^+^-pyrophosphatase and vacuolar V-type H^+^-ATPases generate the driving force for the proton motive force of vacuolar transporter, and P-type H^+^-ATPases generate the driving force for the active transport of K^+^ and Na^+^ (Himabindu et al., 2016). An increase in PM H^+^-ATPase activity by the upregulation of the PM H^+^-ATPase gene imparting salt tolerance was observed in *S. salsa* (Chen et al., 2010). Cloning of ZHA1 from a seagrass (*Zostera marina* L.) depicted a significant expression more in mature leaves toward seawater than the young leaves (Fukuhara et al., 1996). A significantly higher expression of SOS1 was observed in the halophyte *Salicornia dolichostachya* than its glycophyte relative species (*Salicornia oleracea*), playing a dual role in sodium exclusion as well as in xylem loading and unloading. Additionally, Na^+^ could not be retrieved back into the xylem due to the downregulation of HKT1 (Katschnig et al., 2015). In halophytes, the salt stress tolerance mechanism may include preferential K^+^ homeostasis rather than sodium sequestration through membrane-selective antiporters (Kobayashi et al., 2012; Shabala and Pottosin, 2014; Véry et al., 2014). Differential expression of SOS1, SOS2, Na^+^/H^+^ antiporter, cation/H^+^ exchanger, K^+^ efflux antiporter 6-like isoform X2, and plasma membrane Na^+^/H^+^ transporters was observed in the halophytes *U. setulosa* and *Dichanthium annulatum* at salinity levels ranging from EC 30 to 50 dS m^−1^(~300-500 mM of NaCl) (Mann et al., 2019a; Mann et al., 2021b). Under salt stress, key genes for the uptake of NO^3−^, xylem loading of K^+^ and Cl^−^, xylem unloading, and vacuolar compartmentation of Na^+^ and NO^3-^ were upregulated in the roots, while a higher expression of genes for vacuolar K^+^ and Cl^−^ compartmentation was observed in the leaves of *G. inflata*, a versatile industrial and fodder crop of the licorice family (*Glycyrrhiza* spp.) with important medicinal, economic, and forage values (Cai et al., 2023). Salinity and alkali tolerance mechanism, specifically ion homeostasis in *A. lagopoides*, *U. setulosa*, and *Leptochloa fusca*, is correspondingly similar to that of other highly salt-tolerant halophytes, i.e., *T. halophila*, where H^+^-ATPases and Na^+^ and K^+^ transporters are upregulated with high levels of salts (Vera-Estrella et al., 2005; Lata et al., 2019). In *L. ruthenicum*, the expression of LrSOS1 and LrAKT1 increased significantly with NaCl (450 mM) for 6 and 24 h, while that of LrSKOR decreased initially at lower NaCl levels (100 mM) but increased at higher concentrations. Also, LrHKT was downregulated in the roots, but the expression of LrAPV1 and LrNHX was significantly upregulated in the leaves (Hu et al., 2022). De Vos et al. (2019) also reported salt stress-responsive expression of ion transporters and metal transporter proteins in *Artemia*. Root and leaf transcriptomes of *Spartina alterniflora* also revealed a higher expression of genes for ion transporters including Na^+^, K^+^, and Cl^−^ imparting salt tolerance (Bedre et al., 2016). HKT transporters have two subfamilies based on their preference to transport K^+^ and/or Na^+^. Subfamily 1 transporters are selective only for Na^+^ which controls the sodium transport from the roots through the shoots to the leaves (Hauser and Horie, 2010; Suzuki et al., 2016); however, the transporters of subfamily 2 transport both Na^+^ and K^+^ (Corratgé-Faillie et al., 2010; Hmidi et al., 2019). Some candidate genes related to salt bladder or EBC formation were recently identified using transcriptome analysis in *Chenopodium quinoa*, *Mesembryanthemum crystallinum*, and *Atriplex central asiatica* species (Roeurn et al., 2017; Yao et al., 2019).

### 4.2 Transcription factors in salt tolerance

Transcription factors are functional proteins having specific DNA-binding sequences governing transcription (DNA to mRNA) and responsible for the expression of stress-activated genes inducing plant tolerance and adaptation. These transcription factors belong to the families MYB, NAC, bZIP, AP2/ERF, and WRKY extricated in the past playing an important role in metabolic pathways for abiotic stress tolerance (Deinlein et al., 2014; Yoshida et al., 2014; Parihar et al., 2015). In *S. alterniflora*, the transcription factors involved in proton transport also regulate ion homeostasis and, hence, justify their role in salinity stress adaptation (Bedre et al., 2016). Transcriptomics analysis of the halophytes *U. setulosa* and *D. annulatum* at high salt levels revealed major transcription factors (TFs) belonging to bZIP, WRKY, BTB/POZ, MYB, MADS, DREB, AP2-EREBP, NAC, bHLH, and SBP, where MYB TFs were highly expressed (Mann et al., 2021b). These Myb and WRKY transcription factors have an active role in drought stress tolerance and signaling as well (Banerjee and Roychoudhury, 2015; Li et al., 2015). The differential regulation of transcription factors such as WRKY was seen in *S. fruticosa* for tolerance to abiotic stress (Diray-Arce et al., 2015). Other TFs, namely, bZip (basic region leucine zipper) proteins, zinc finger proteins, homeobox proteins, MADS domain proteins, and basic helix-loop-helix (bHLH) encoding genes, positively regulate salt stress signals in *Arabidopsis* and other halophytes imparting salt and osmotic stress tolerance (Shen et al., 2003; Zhou et al., 2009; Zhang et al., 2016). Introgression of the NAC transcription factor AINAC4 from *A. lagopoides* into tobacco plants enhanced salt tolerance through the upregulated activities of antioxidant enzymes (Khedia et al., 2018). Similarly, differential expression of MYB, CAMTA, MADS-box, and bZIP TFs depicted the salt tolerance of *S. fruticosa* (Diray-Arce et al., 2019). Vast information on the role of DREB genes in improving salt tolerance is available in many crop plants.

### 4.3 MicroRNAs

The role of single-stranded, 21- to 23-nucleotide-long, non-coding RNA molecules has been revealed in post-transcriptional gene regulation and gene silencing under various environmental stresses, more specifically in glycophytes. Although few salt-responsive microRNAs (miRNAs) and their targets have been validated through NGS and gene expression analysis, the mining of post-transcriptional regulation of gene expression in halophytes by miRNA is still in its infancy. These miRNA targets might function through differential expression of certain genes, structural changes in proteins, and/or generation of secondary metabolites. An expression kinetic study on the halophyte *S. alterniflora* Loisel reported the differential expression of 12 miRNAs in the roots as well as leaf tissues under salinity (Zandkarimi et al., 2015). Furthermore, gene expression analysis of putative novel microRNAs advocated that halophytes may possess post-transcriptional gene regulation for adaptation to salinity stress. Similarly, in *S. maritima*, the reactivity of miRNAs (sma-miR2 and sma-miR) to salinity stress discloses the role of tiny single-stranded RNA sequence imparting tolerance (Gharat and Shaw, 2015) against seawater. Notably, the role of miRNA in the adaptation of the highly salt-tolerant *Oryza coarctata* (a wild relative of *Oryza sativa*) has been published, which unwinds new avenues for rice researchers to develop salt-tolerant rice cultivars (Mondal et al., 2015; Parmar et al., 2020). miRNAs for enzymes involved in lignin biosynthesis, carbon and nitrogen metabolism, transcription factors, and nucleotide binding site-leucine-rich repeat proteins were identified in *S. europaea* in response to salt stress (Feng et al., 2015). miR414 and miR5658 were identified from *Tamarix* providing post-transcriptional salt stress responses (Wang et al., 2018).

## 5 Proteomic regulation

The study of whole-body proteins in any organism is termed as proteomics (Zhang et al., 2012a), which can be used to decode proteomic information at the cell, tissue, organ, or plant level. A number of proteins work in several networking pathways to provide salt stress tolerance such as stress/defense-induced proteins, e.g., ROS scavenging enzymes against oxidative stress; pathogenesis-related proteins; osmotin against osmotic stress; proteins involved in photosynthesis, redox homeostasis, carbohydrate and energy metabolism (ATP synthesis, photosynthesis, and respiration), signal transduction, and protein and lipid metabolism; cytoskeleton and cytoskeleton-associated proteins; proteins involved in the metabolism of osmolytes and phytohormones and membrane transport; and enzymes involved in lignin biosynthesis or cyanate degradation (Parihar et al., 2015). The proteomic analysis in *P. tenuiflora* revealed the differential expression of structural proteins, e.g., D1 protein for assembling and stability of PSII was downregulated (Yu et al., 2011); however, upregulation of D2 protein was reported in *A. lagopoides* (Sobhanian et al., 2010). The photosynthetic enzymes, i.e., ribulose-bisphosphate carboxylase small chain and ferredoxin, were found to be upregulated in response to high salt concentration in *D. annulatum* and *U. setulosa* (Mann et al., 2019a; Mann et al., 2021b) similar to that observed in *Physcomitrella patens* gametophyte (Wang et al., 2008) and in *Porteresia coarctata* leaves (Sengupta and Majumder, 2009). Conversely, decreased expression of the thylakoid lumen (TL) proteins, i.e., TL19 and TL18.3, has been reported in *A. thaliana* and *T. halophila*, respectively (Zhang et al., 2012b). The comparative proteomic analysis of the halophyte *Tangut nitraria* revealed altered expressions of 71 proteins when exposed to salinity stress (Cheng et al., 2015). Proteomic expression of the halophyte *H. glomeratus* identified 87 salt-responsive proteins (Wang et al., 2015; Wang et al., 2016) playing key roles in signal transduction; stress defense; carbohydrate, protein, and energy metabolism; cytoskeleton metabolism; and cell growth. Salt stress-responsive proteins have been identified through proteomic analysis of various halophytes including *S. aegyptiaca* (Askari et al., 2006), *Bruguiera gymnorrhiza* (Tada and Kashimura, 2009), *S. europaea* (Wang et al., 2009; Fan et al., 2011), *A. lagopoides* (Sobhanian et al., 2010), *Cakile maritima* (Debez et al., 2012), *P. tenuiflora* (Yu et al., 2011), *T. halophila* (Wang et al., 2013), *K. candel*, *H. glomeratus* (Wang et al., 2015; Wang et al., 2015), and *Atriplex griffithii* (Jha, 2022) regulating signal transduction, ionic homeostasis, photosynthesis, ROS scavenging, etc. under salt stress conditions.

## 6 Halophytic genes as a source of salt tolerance

The survival capability of halophytes from moderate to extreme saline soils enables them to be explored deeply up to the gene and gene network level. Emerging technologies such as high-throughput RNA sequencing technologies have paved the way easily to translate genetic information into salt stress response and further downstream signaling for better adaptive strategies. The advantage of RNA sequencing is that the transcriptome of one species can be successfully used to computationally predict the miRNAs in other related species having a similar metabolism, even if they belong to a separate taxonomy. Glycophytes are being compared with halophytes through RNA sequencing to pinpoint the most important pathways for survival and tolerance at various salinity levels. Although many salt-responsive genes from *Arabidopsis* have been cloned and overexpressed in many crop plants for enhanced salt tolerance, a few genes from halophytes have also been transferred in crop plants with better tolerance limits. For example, overexpression of halophytic enzymes in glycophytic plants involved in the synthesis of osmoprotectants (glycine betaine, raffinose, choline monooxygenase, betaine aldehyde dehydrogenase, and galactinol synthase) led to increased salt tolerance (Wu et al., 2010; Sun et al., 2013). Overexpression of OsCPK4 enhanced salt tolerance in rice (Campo et al., 2014). Similarly, overexpression of vacuolar ATPase from the halophyte *S. alterniflora* in rice enhanced salt tolerance through increased K^+^/Na^+^ ratio, ABA-mediated stomatal closure, and high water content (Baisakh et al., 2012). Transgenic plants of *Jatropha curcas* with the SbNHX1 gene from the halophyte *S. brachiata* were developed with enhanced salt tolerance up to 200 mM of NaCl (Joshi et al., 2013). Some novel genes such as *USP*, *SDR1*, and *SRP* along with genes for ion channels (Cl^−^, Ca^2+^, aquaporins), antioxidant encoding genes (*APX*, *CAT*, *GST*, *BADH*, *SOD*), and antiporter genes (*NHX*, *SOS*, *HKT*, *VTPase*) were isolated from halophytes and transferred to glycophytes to enhance stress tolerance (Mishra and Tanna, 2017). Saad et al. (2011) have identified a stress-responsive tissue-specific promoter, *PrAlSAP*, a plant age-dependent candidate gene from a C4 halophyte grass (*Aeluropus littoralis*), to be used for enhancing salt stress tolerance of glycophytes. Differentially expressed genes (DEGs) were also identified for Na^+^ compartmentalization, Ca^+^ enrichment, and ABA signaling in *Salicornia persica* through gene network analysis (Aliakbari et al., 2020). Furthermore, the halophytic SOS1 (*PtNHA1*) and NHX (*PutNHX*) antiporter genes were cloned from *P. tenuiflora* and transferred to produce genetically engineered salt-tolerant rice plants (Kobayashi et al., 2012) where the transgenic plants maintained low Na^+^ and high K^+^ in the shoots under salinity. Similarly, transgenic rice exhibited tolerance up to 300 mM of NaCl after transformation with the vacuolar antiporter NHX1 from the halophytes *Atriplex gmelinii* (Ohta et al., 2002), *S. salsa* (Zhao and Zhang, 2006), and *Spartina anglica* (Lan et al., 2011). The complete coding sequence of the *Dehydrin* gene from the halophyte *U. setulosa* was cloned in pCAMBIA1304 binary vector and transformed in rice through *Agrobacterium*. Three T0 plants positive for the HPTII gene were identified and T1 seeds were harvested (Mann et al., unpublished data). These transgenes need further functional validation. A brief compiled list of halophytic genes overexpressed or cloned for enhanced salt tolerance in various plant systems is provided in **Table 2**. The other genes used for enhanced salt tolerance were for osmoregulation (Das-Chatterjee et al., 2006; Wang et al., 2013), increased photosynthetic efficiency (Prashanth et al., 2008), ion partitioning (Mahalakshmi et al., 2006), better ROS scavenging (Zhang et al., 2014), and maintaining cell turgor (Singh et al., 2014). Overexpression of PvLBD12 enhanced salt tolerance by altering a wide range of physiological responses like increased proline accumulation, reduced malondialdehyde production, improved K^+^ accumulation, and reduced Na^+^ absorption in switchgrass (*Panicum virgatum* L.) (Guan et al., 2023). Recently, the antiporter *NsNHX1* gene from the halophyte *Nitraria sibirica* has been used for better plant growth and salinity tolerance in the tree species poplar through improved ion homeostasis, osmoregulation, increased photosynthetic efficiency (Geng et al., 2020) and, hence, better tree growth under salinity. Thus, halophytes can be explored to enhance the salt stress tolerance of plant species in a broader way.

**Table 2:** Halophytic genes as sources for enhanced salt tolerance in crop plants.

Sr. no.	Gene name	Source of the gene	Plant used	Adaptive salt tolerance strategy	References
1.	*AgNHX1*	*Atriplex gmelini*	Rice	Enhanced vacuolar antiporter activity in transgenic rice	Ohta et al. (2002)
2.	*AeNHX1*	*Agropyron elongatum*	*Arabidopsis*, *Festuca*	Improved salt tolerance through better osmotic adjustment and efficient photosynthesis	Qiao et al. (2007)
3.	*AlNHX*	*Aeluropus littoralis*	Tobacco, soybean	Higher K^+^/Na^+^ ratio in the leaves	Zhang et al. (2008); Liu et al. (2014)
4.	*HcNHX1 HcVP1 HcVHA-B*	*Halostachy scaspica*	*Arabidopsis*	Better seed germination Na^+^ compartmentalization in the leaves	Guan et al. (2011); Hu et al. (2012)
5.	*KfVP1*	*Kalidium foliatum*	*Arabidopsis*	Na^+^ compartmentalization in the leaves	Yao et al. (2012)
6.	*KcNHX1/KcNHX2*	*Karelinia caspica*	*Karelinia caspica*	Silencing of this gene through RNAi decreased salt tolerance which further improved salt tolerance in transgenics	Liu et al. (2012)
7.	*LfNHX1*	*Leptochloa fusca*	Tobacco	Better germination and root growth, increased salt tolerance	Rauf et al. (2014)
8.	*PhaHAK2*	*Phragmites australis*	Reed plant	Higher K^+^/Na^+^ ratio in the leaves with more uptake of K^+^	Takahashi et al. (2007)
9.	*PeNHX1–6*	*Populus euphratica*	Onion	Compensated Na^+^/H^+^ antiporter activity for salt tolerance	Ye et al. (2009); Wang et al. (2014)
10.	*PtNHA1 and PutNHX PutHKT2; 1 PutAKT1 KPutB1*	*Puccinellia tenuiflora*	Rice, *Arabidopsis*	Shoots were more tolerant to NaCl and roots showed tolerance to Na_2_CO_3_ Higher K^+^/Na^+^ ratio in the leaves	Kobayashi et al. (2012); Ardie et al. (2009); Ardie et al. (2010); Ardie et al. (2011)
11.	*SbNHX1*	*Salicornia brachiata*	Tobacco, *Jatropha*	Increased salt tolerance in transgenics	Jha et al. (2011); Joshi et al. (2013)
12.	*SeNHX1*	*Salicornia europaea*	Tobacco, alfalfa	Increased osmolytes, e.g., betaine in tobacco and proline in alfalfa	Zhou et al. (2008); Zhang et al. (2014); Chen et al. (2015a)
13.	*SsNHX1*	*Salsola soda*	Alfalfa	Enhanced tolerance up to 400 mM of NaCl and Na^+^ sequestration into vacuole	Li et al. (2011)
14.	*SaVHAc1*	*Spartina alterniflora*	Rice	Improved salt tolerance, upregulation of DEGs for cation transport and ABA signaling	Baisakh et al. (2012)
15.	*SaNHX1*	*Spartina anglica*	Rice	Enhanced salt tolerance in transgenic rice	Lan et al. (2011)
16.	*SsNHX1 SsVP SsHKT1; 1 SsDREB*	*Suaeda salsa*	Rice, *Arabidopsis*, tobacco	Increased salt tolerance in transgenic plants Potassium selectivity for salt tolerance	Zhao et al. (2009); Guo et al. (2006); Shao et al. (2008); Hongbo et al. (2015)
17.	*ScVP ScNHX1 ScVP*	*Suaeda corniculata*	*Arabidopsis*, alfalfa	Na^+^ ion partitioning in the leaves and roots	Liu et al. (2011); Liu et al. (2013)
18.	*ThNHX1*	*Thellungiella halophila*	*Arabidopsis*	Increased salt tolerance of transgenics	Wu et al. (2009)
19.	*TsVP TsHKT1; 2*	*Thellungiella halophila*	Tobacco, cotton	Improved salt tolerance in tobacco due to ion compartmentalization of Na^+^ in vacuoles Accumulation of Na^+^ and Cl^−^ in vacuoles and osmoregulation in cotton	Gao et al. (2006); Lv et al. (2008)
20.	*ZmVP1*	*Zoysia matrella*	*Arabidopsis*	Sodium sequestration in vacuole	Chen et al. (2015b)
21.	*AoDREB*	*Asparagus officinalis*	*Arabidopsis*	Improved salt tolerance	Liu et al. (2010)

### 6.1 Major constraints for halophytes as donors

Halophytes are being explored deeply for their survival and tolerance mechanism under saline conditions through physiological, biochemical, and molecular studies or even using RNA sequencing or proteomics studies. Still, the precise information on their pathways defining salt tolerance mechanisms needs to be charted in detail. Differentially expressed genes or proteins have been identified and cloned into host plants for enhanced salt tolerance using genes for vacuolar or plasma membrane Na^+^/H^+^ antiporters, potassium transporters, vacuolar pyrophosphatase, antioxidants, WRKYs, and proteins involved in signal transduction and defense mechanism. Many reports have proved halophytes as one of the most appropriate models for studying different salt stress tolerance mechanisms (Shabala, 2013; Flowers et al., 2015; Himabindu et al., 2016). Still, there are many difficulties that need to be overcome for using halophytes in the agriculture system. One of the major problems is seed germination directly in saline conditions; others include the propagation of plants and even genotype selection due to less genetic variation among related species (Ventura et al., 2015). Additionally, introducing non-domesticated halophytic plant species as agricultural crops with reasonable income for the growers requires refinement of growing protocols and selection of improved varieties. During the 1960s, Nurock (1960) and his team initiated a 6-year plant introduction program for the development of agricultural and pasture material suitable for dryland conditions. This initiation led to the development of the first milestone for a plant collection of xerophytes and halophytes in the form of an ecological desert garden (Boyko, 1962). Also, the lack of recommended cultivation protocols for halophyte crops limits their use in comparison to conventional crops. However, Zerai et al. (2010) proposed a breeding program for selection based on yield parameters in *Salicornia bigelovii.* For the sustainability of saline agricultural practices, the correct choice of adequate cultivation systems for different types of halophytes is of utmost importance. The coastal halophytes require different practices than those of the dryland regions. The halophytic crops should also undergo the same process as the conventional agricultural crops including breeding for the improvement of agricultural traits such as yield and taste and mechanical harvesting over a stable time period to refine their growing habit, seed availability and stability of genetic traits, and generation advancement to be used in crop improvement programs. Despite many limitations, the use of halophytes for the development of transgenics with enhanced salt tolerance is in progress. The major favorable parameters for halophytes in comparison to crop plants are their least management, low-cost ratio, easier harvesting, and better seed viability. A range of cultivation systems for the utilization of halophytes have been developed for the production of biofuel, purification of saline effluent in constructed wetlands, landscaping, cultivation of gourmet vegetables, and more for their long-term use.

## 7 Economic potential of halophytes

A wide range of halophytes are used in various ecological zones of India for food, feed, biofuel, medicine, raw materials for industrial use, etc. (Dagar, 2003). The utilization of the most noxious weed *Alhagi maurorum*, thriving excellently on saline, sandy, rocky, and dry soils, as a soil ameliorant was the oldest known attempt to employ halophytic plants. The major parts of productive farmlands are turned into wastelands over the coastal region due to severe wave conditions in the Bay of Bengal. Here, halophytes help to utilize the waste coastal land for agricultural use. To reduce post-cyclone stress, deep furrow and high ridge shaping techniques were developed on Sundarbans coastal land (Bandyopadhyay et al., 2008). A land-shaping method was created by converting land into shallow furrows and medium ridges, where suitable halophytic trees were grown together with paddy–cum–fish cultivation during the kharif season, and furrows were utilized for rice cultivation in the rabi season (Burman et al., 2013).

Halophytes are being prioritized for their contribution toward saline land reclamation, environmental protection, and food and animal feed security, with their primary utility in traditional societies as sources of drugs. The lignocellulosic biomass of four halophytes, namely, *Pongamia*, *Jatropha*, *P. virgatum*, and *Miscanthus*, is one of the four main raw materials utilized for the manufacturing of second-generation biofuel in India. In addition, halophytes are valuable sources of nutraceuticals and exclusive sources of traditional medicine. Halophytes are also being recognized for their nutritional, medicinal, and economic values mainly due to phenolics, flavonoids, and secondary metabolites as compared with the normal vegetation in other climatic regions. The accumulation of functionally important secondary metabolites is one of the defensive mechanisms used by plants to cope with environmental abiotic stress (Borges et al., 2017; Dresselhaus and Hückelhoven, 2018). The plant metabolites that are produced in response to environmental and other plant defense-related stress factors have shown distinct biological effectiveness and therapeutic potential against communicable and non-communicable diseases, such as microbial infections, oxidative stress, and related disorders, as well as against cancer (Stankovic et al., 2019; Lopes et al., 2021). Halophytes are also a potential source of other secondary metabolites, including alkaloids, saponins, iridoids, sterols, terpenoids, volatile oils, and certain bitter principles (Faustino et al., 2019; Mohammed et al., 2023). The halophytes and other plants used in traditional medicines have served populations dwelling in far-reaching areas where modern medicine and its facilities have not penetrated. Halophytes have, for a long time, been the crucial component of traditional herbal medicine, and in this capacity, they have also served the nomadic tribes in the Arabian Desert (Ksouri et al., 2012; Giordano et al., 2021). Nonetheless, the range of bioactivity of halophytes covers a broader segment in disease amelioration and includes plants showing antibacterial, antifungal, anticancer, and antiviral properties. Halophytes are also used to treat chronic diseases of the liver, heart, and kidneys, including jaundice, hypertension, diabetes, renal insufficiency, and renal calculi, by local and nomadic tribes in various regions where they occur (Mohammed et al., 2023). Halophytes have historically been employed in traditional medicine by villagers, and very little documentation is available on this aspect. For example, the oil from the mangrove *Cynometra ramiflora* is used to treat skin conditions and contains antibacterial qualities. *Salvadora persica*, *Salsola baryosma*, and *S. kali* as an anthelmintic, emmenagogue, and diuretic; *Tamarix articulata* used in eczema, ulcers, piles, sore throat, diarrhea, and liver disorders; and *Cress cretica* as a tonic, aphrodisiac, and stomachic deserve special mention among other notable uses of halophytes as medicine (Abeysinghe, 2010). Sulfated polysaccharide harvested from seaweed can be a potent molecule to fight against the COVID-19 pandemic; hence, it is a candidate molecule to be studied against SARS-CoV-2 (Jha et al., 2020).

## 8 Summary and future prospects

The available knowledge on halophytes and their salt tolerance mechanisms has been used in generating a reliable database, and overexpression of related genes enhanced salt tolerance in related crop plants. The salt tolerance mechanism is, moreover, similar in halophytes with a variable degree and intensity of expression initiating with the reception of salt ions through the SOS system; ion sequestration and compartmentalization; selective membrane transporters; osmoregulation and down signaling through various metabolites; relative expression of genes, TFs, and proteins to counteract the adverse effects; and imparting tolerance for better plant growth and survival. Earlier reports have invariably compiled information about the transfer of halophytic genes into non-plant systems, but herein, we have focused on and reviewed their role in crop plants. Since gene transformation studies have been quite successful for enhanced tolerance limits in crop plants, in that case, through this vast information, our hypothesis is why these halophytes cannot be used as model plant species for salt tolerance studies at the field scale for real ground truthing of built-in tolerance mechanisms and adaptation strategies. The recent advent of the CRISPR/Cas9 system has further logged up the genetic engineering in plants for desired traits, and herein, halophytes are best suited for gene editing for salinity or drought adaptability. Future research on halophytes would be helpful to develop crop varieties that can withstand low moisture and salt stress environments.

## Author contributions

AM and AK conceived and designed the research topic. ArK performed data analysis. CL and NK prepared rough draft of manuscript and performed the experiments. PS edited the manuscript. All authors contributed to the article and approved the submitted version.
